# Associations of chronotype and socio-demographic factors with timing of eating in finnish preschool-aged children

**DOI:** 10.1007/s00394-025-03800-z

**Published:** 2025-09-18

**Authors:** Ilse Tillman, Mirkka Maukonen, Anna M. Ruokolahti, Henna Vepsäläinen, Carola Ray, Jenna Rahkola, Eva Roos, Maijaliisa Erkkola, Reetta Lehto

**Affiliations:** 1 Department of Food and Nutrition, University of Helsinki, Helsinki, Finland; 2 Folkhälsan Research Center, Helsinki, Finland; 3 Finnish Institute for Health and Welfare, Helsinki, Finland; 4 Department of Food Studies, Nutrition and Dietetics, Uppsala University, Uppsala, Sweden; 5 Department of Public Health, University of Helsinki, Helsinki, Finland

**Keywords:** Chrononutrition, Food timing, Dietary patterns, Temporal eating patterns, Circadian rhythm

## Abstract

**Purpose:**

Chrononutrition, encompassing timing, frequency, and regularity of dietary intake, may affect metabolic health and chronic illness risk, making early dietary patterns crucial. This study aimed to explore potential determinants of chrononutrition among preschoolers.

**Methods:**

The cross-sectional DAGIS study included 677 Finnish preschoolers aged 3–6. Data were collected through 3-day food records and 7-day actigraphy-measured sleep. ANCOVA and linear regression were used to analyze associations between potential determinants—chronotype, SES, parental work hours, age, and sex—and chrononutrition variables (timing of the first and last EOs, energy and eating midpoints, duration of the fasting window, morning and evening latency, and the number of EOs).

**Results:**

A later chronotype was associated with later timing for first and last EOs, eating and energy midpoints (*p* < 0.001), shorter morning (*p* = 0.002), and longer evening latency (*p* < 0.001). Children whose fathers worked regular hours had a longer fasting window compared to children whose fathers did not work (*p* = 0.03), and a longer morning latency compared to children whose fathers did shift work (*p* = 0.04). High SES was associated with later energy midpoint (*p* = 0.004). On weekdays children whose mothers worked regular hours had their first EO earlier compared to children whose mothers worked shifts (*p* = 0.006) and a shorter fasting window (*p* = 0.009). During weekend days boys had a longer morning latency compared to girls (*p* < 0.001), and children with morning (*p* = 0.006) and intermediate (*p* = 0.02) chronotypes had more EOs compared to evening chronotypes.

**Conclusion:**

Chronotype was a key determinant of the timing of food intake in Finnish preschool-aged children, while sociodemographic factors had a less pronounced association.

**Supplementary Information:**

The online version contains supplementary material available at 10.1007/s00394-025-03800-z.

## Introduction

Early childhood years are crucial for developing lifelong eating patterns [1]. While research has primarily focused on factors affecting what children eat, the concept of chrononutrition—encompassing the timing, frequency, and regularity of food intake in relation to the sleep‒wake cycle, has received less attention [2–4]. Chrononutrition is crucial, as misalignment between food intake and the body’s inner circadian rhythm can disrupt metabolic health, causing elevated levels of insulin, glucose, and inflammatory markers, which can increase the risk for various adverse health outcomes, including cardiovascular disease, obesity and type 2 diabetes [2, 5, 6].

Several chrononutrition variables have been recognized as potentially associated with metabolic health outcomes. The definition of an eating occasion (EO) is typically based on time intervals and/or the energy content consumed [7]. Research has shown that the first and last EO of the day can advance or delay the phase of the inner clock and therefore help synchronize the body’s circadian rhythm [8]. Eating midpoint is defined as the clock time halfway between the first and last EO of the day, while energy midpoint indicates the time of day when 50% of the daily calories are consumed [9]. Findings from a recent systematic review of intervention trials by Young et al. showed that a later distribution of energy intake was associated with reduced insulin sensitivity and increased weight in adults [10]. Other reviews, focusing on studies in adults and animals, showed that prolonged (> 12 h) fasting windows—the period between the last meal of the day and the first meal the following day—had beneficial changes in insulin sensitivity and glucose metabolism [11] and may contribute to weight loss [4]. Morning latency refers to the interval between waking up and the first EO of the day, usually breakfast [1]. The first EO is critical for setting the tone for food intake for the rest of the day, as research has shown that adults who eat breakfast tend to have better diet quality and better regulation of their overall energy intake [12]. Skipping breakfast, on the other hand, has been associated with an increased risk of overweight and obesity in children [3]. Not only does the timing of EOs differ between individuals, but also the frequency. In children, a higher daily number of EOs has been associated with lower total and LDL cholesterol, higher diet quality, and lower level of overweight [3, 13, 14].

Chronotype reflects an individual’s circadian rhythm, aligning the internal biological clock with the 24-h day [15]. It ranges from extreme early to late types [16] and is influenced by genetics, environmental cues like light exposure, social schedules, sex, and age, making it dynamic [2, 15]. Chronotype tends to shift toward eveningness in adolescence and reverts toward morningness in adulthood [17]. Assessment can be done through the Morningness-Eveningness Questionnaire (MEQ) for preferred sleep and activity times [18], or by evaluating midpoint of sleep on free days adjusted for sleep debt using either the self-reported Munich ChronoType Questionnaire (MCTQ) [17] or actigraphy [19]. Biological markers such as melatonin onset in dim light can also be used [15, 20]. Chronotypes are generally categorized as morning, intermediate or evening, with most individuals being intermediate chronotypes [17].

A review by Dashti et al. [21] found that chronotype, sex, age, socioeconomic status (SES), and work hours are all potential determinants for food timing in adults. Research on determinants regarding chrononutrition variables in children remains notably underexplored [2], and most studies have focused on the parental influence of breakfast skipping [1, 22]. Chronotype has been linked to the timing of food intake in Asian school-aged children [23, 24]. Children with a morning chronotype were associated with earlier timing for breakfast, lunch and dinner [23] and also had breakfast more regularly than children with an evening chronotype [24]. Sociodemographic factors at the parental level are also potential determinants of children’s chrononutrition patterns. Working parents often miss meals and have rushed mealtimes [25, 26]. Children with at least one parent working nonstandard hours are less likely to eat breakfast [22], suggesting that parents’ work hours can disrupt family meal routines. Higher SES among parents has been associated with a greater likelihood of children consuming breakfast [27, 28]. Sex-related differences in chrononutrition factors have not been studied in young children; however, research indicates that general eating patterns in children under 6-years old are similar across boys and girls [29, 30]. The average number of EOs usually decreases while portion size increases with age during preschool years [31, 32]. Furthermore, there is a substantial lack of research regarding variability in the timing of food intake between weekdays and weekends in young children. A study on Swedish preschool-aged children found that they had fewer EOs on days not attending preschool [33], and another study on primary school-aged children in China found that evening-type children had less consistent meal timing between weekdays and weekends compared to children with a morning chronotype [23].

Since the timing of food intake may affect metabolism [5, 34] and the eating patterns formed in childhood persists into adulthood [2], understanding the determinants of children’s chrononutrition patterns is crucial for developing dietary strategies to prevent diet-related chronic diseases later in life [35]. There is a significant gap in the literature regarding the factors influencing individual variability in food timing among young children. This study aimed to investigate how chronotype tendency and sociodemographic factors such as SES, sex, and age associate with chrononutrition patterns in preschool-aged children, and if parents’ irregular work hours are associated with the timing of food intake. Additionally, we examined the potential associations separately for weekdays and weekends.

## Participants and methods

### Participants

The participants were drawn from the Increased Health and Wellbeing in Early Education and School Children (DAGIS) survey. The survey was conducted between September 2015 and April 2016 in 66 early childhood education and care (ECEC) centers situated in southern and western Finland and is described in more detail elsewhere [36]. A total of 864 preschoolers (24% of those invited) aged 3–6 years participated in the DAGIS study, of whom 677 (78%) were included in the current study. 175 (20%) of the original participants were excluded due to having less than 3 days of food records, or because the dates from the food records did not match the dates from their sleep data, and 12 (1%) due to having less than 7 nights of sleep records. The DAGIS study was approved by the University of Helsinki, Research Ethics Committee in the Humanities and Social and Behavioral Sciences 2/2015 (#6/2015).

### Food records and sleep data

A combination of food records and actigraph-measured sleep data collected on the same dates was used to create the chrononutrition variables. 3-day food records containing two weekdays and one weekend day, including food quantities and timing, were completed by the ECEC staff during preschool hours and by parents of the participants at home. 40% of the participants documented their food intake on consecutive days, while the remaining participants recorded non-consecutive days. Share of weekdays and weekend days recorded did not differ between the two approaches. Portion sizes were estimated with the help of a validated Children’s Food Picture Book [37]. The food record data collected were entered into AivoDiet dietary software, where energy intake was calculated for the analyses. Further details about the collection and handling of the food record data have been described by Korkalo et al. [38]. Sleep was measured with a hip-worn ActiGraph wGT3X-BT triaxial accelerometer (Pensacola, FL, USA) for 7 consecutive days, 24 h per day except during water-based activities such as taking a swim or bath. Less than 16 h of wear from a noon-to-noon window was considered invalid. Weekend nights were defined as the nights between Friday and Saturday and between Saturday and Sunday. Additional details about the actigraph data collection and processing have previously been described elsewhere [39].

### Chrononutrition variables

The study focused on the following chrononutrition variables derived from the food and sleep records: (i) timing of the first EO after waking up in the morning, and (ii) the last EO before falling asleep at night, (iii) duration of the fasting window, (iv) number of EOs, (v) duration of morning and (vi) evening latency as well as (vii) the timing of eating and (viii) energy midpoints. An EO was defined in accordance with earlier research [14, 40] as food and beverages recorded within 15 min. EOs containing less than 210 kJ (6.7% of total entries) were excluded. The fasting window was defined as the time elapsed between the last EO at night and the first EO the next day. Morning latency was calculated by subtracting the wake-up time from the time of the first EO, and evening latency was calculated by subtracting the time of the last EO from the time the participant fell asleep. All included participants provided 3-day food records with corresponding sleep data. However, if a participant lacked complete food records for both evening latency and morning latency on the following day, the corresponding variable was classified as missing (NA) for that instance. Eating midpoint was determined as the mid clock time between the first and last EOs within a single day, and energy midpoint was defined as the time of day when half of the total energy intake was reached. For all variables, separate counts were made for all 3 days as an average as well as for weekdays and weekends separately. For all chrononutrition variables, except the number of EOs, weekends were defined as spanning from 18:00 on Fridays to 17:59 on Sundays. This definition aligns with the transition from weekday routines to weekend behaviors, capturing changes in timing patterns that may occur as individuals shift away from a structured weekday schedule**.** For the analysis of the number of EOs, Saturday and Sunday were classified as weekend days, while Monday through Friday were considered weekdays, reflecting the structured routine associated with preschool attendance during the week.

### Potential determinants of chrononutrition

Sociodemographic data, sex (boy/girl), and date of birth were obtained through a questionnaire filled out by parents. Age was calculated by subtracting the birthday from the research date, and the children were based on midpoint divided into two groups, 3–4 yrs and 5–6 yrs old. The highest parental educational level in the family was used as an indicator for SES. The level of education ranged from comprehensive school to a doctoral level degree. Responses were categorized for analyses into three groups: (1) low SES (high school degree, vocational diploma, or less), (2) medium SES (associate or bachelor’s degree), and (3) high SES (master’s, licentiate, or doctorate degree). Parents were asked to provide information about their work hours, resulting in the following three categories: (1) Regular working hours (regular daytime hours (6–18)), (2) shift work (regular night work, fixed and rotating two- and three-shift patterns), and (3) do not work (not employed). Participants who checked alternatives that did not conform to the classification schemes (seasonal work, work only on weekends, other working arrangements, part-time jobs, and entrepreneur) or had missing information were excluded from that particular analysis. The formula by Roenneberg et al. [17] was applied to calculate continuous chronotype tendency as follows: *mean midpoint of weekend sleep—(mean weekend sleep duration—mean weekly sleep duration) / 2*. If sleep duration was longer on weekends, the midpoint of sleep was adjusted for potential sleep debt accumulated during the weeknights. In cases where sleep duration was not longer on weekends, the weekend midpoint value was used directly. None of the children attended preschool on weekends; therefore, it was assumed that weekend sleep (Friday-Saturday, Saturday-Sunday) was free from a set schedule. Categorical chronotype tendency was determined using a conservative method based on prior studies in similarly aged children reporting the prevalence of children with evening chronotype being 10–26% [41–44]. Children in the lowest 10th percentile were classified as having a morning chronotype tendency, those in the highest 10th percentile as having an evening chronotype tendency, and those between the 10 and 90th percentiles as having an intermediate chronotype tendency.

### Statistical analysis

Before conducting the analyses, the assumptions underlying each statistical method were systematically checked. For one-way analysis of variance (ANOVA), normality was visually assessed using Q-Q plots, and the homogeneity of variance was evaluated using the Breusch-Pagan test. For analyses of covariance (ANCOVA) and multivariate linear regression, assumptions regarding linearity and homoscedasticity were visually examined through residual plots, while the absence of multicollinearity among predictor variables was assessed using the Variance Inflation Factor (VIF). Additionally, potential outliers were identified and examined to ensure robust results. Addressing these assumptions strengthened the validity of the statistical findings and the conclusions drawn from the analysis.

The associations between each independent determinant (chronotype tendency, SES, parents’ work hours, age, sex) and the chrononutrition variables (first and last EO, fasting window, morning and evening latency, eating and energy midpoint, number of EOs) were assessed via ANOVA. To preserve the full range of individual variability regarding continuous determinants (chronotype and age), univariate linear regression was conducted to test for associations with the chrononutrition variables. To produce results for each potential determinant’s relationship with the chrononutrition variables independent of the other determinants, ANCOVAs with all independent determinants included simultaneously were applied. Tukey’s post-hoc test was performed for comparisons between subgroups following significant associations from the ANCOVAs. Multivariate linear regression was carried out for continuous chronotype and age, with all independent determinants incorporated concurrently, enabling the examination of both continuous variables’ independent relationships with the chrononutrition variables.

All analyses were conducted using Statistical Software R version 4.4.1. Results with *p* < 0.05 were considered statistically significant.

## Results

### Sample characteristics

The characteristics of the study sample are presented in Table 1. Of the 677 children included in the study, 53% were male. The overall mean age was 4.7 years (SD 0.9). The younger age group had a mean age of 4.1 years (SD 0.6), while the older age group had a mean age of 5.5 years (SD 0.5). The middle SES was most prevalent (42%) and most mothers (71%), and fathers (82%) worked regular hours.

**Table 1:** Demographic characteristics of the study sample from the DAGIS study, 2015–2016

	n (%)
Age	677 (100)
3–4 yrs	383 (57)
5–6 yrs	294 (43)
Sex	677 (100)
Boys	360 (53)
Girls	317 (47)
Chronotype tendency	677 (100)
Morning	70 (10)
Intermediate	540 (80)
Evening	67 (10)
Mother’s work hours	598 (88)
Regular	424 (71)
Shift	84 (14)
Do not work	90 (15)
Missing	79 (12)
Father’s work hours	539 (80)
Regular	441 (82)
Shift	73 (14)
Do not work	25 (5)
Missing	138 (20)
SES	675 (99.7)
Low	147 (22)
Middle	281 (42)
High	247 (36)

### Associations between potential determinants and chrononutrition variables

#### Three-day mean values

**Fig. 1:**
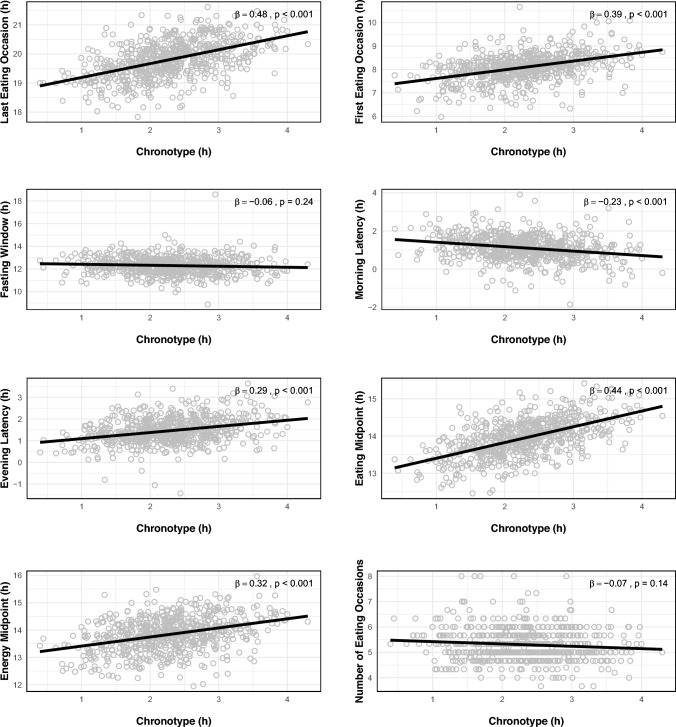
Multivariate regression plots showing associations between continuous standardized chronotype and chrononutrition variables. Plots were generated with R version 4.4.1, utilizing packages openxlsx, broom, ggplot2, gridExtra, and grid

Children exhibiting a morning chronotype tendency displayed the earliest clock times for both the first and last EOs, along with their eating and energy midpoints (Table 2). In contrast, children with an evening chronotype tendency showed the latest timings. These differences were statistically significant across all chronotype tendency groups (*p* < 0.001). The difference in timing between morning and evening chronotype tendency groups for the first EO was 61 min (7:35 vs. 8:36), and for the last EO was also 61 min (19:17 vs. 20:18), indicating no significant difference in the duration of the fasting window (*p* = 0.98). Additionally, children with a morning chronotype tendency demonstrated a shorter evening latency (61 min) compared to those with evening chronotype tendency (108 min) (*p* < 0.001), while their morning latency (79 min) was longer compared to children with intermediate (62 min) (*p* = 0.01) and evening chronotype tendencies (52 min) (*p* = 0.002). However, no significant difference in morning latency was found between children with intermediate and evening chronotype tendencies (*p* = 0.19). Furthermore, there were no significant differences in the number of EOs across chronotype tendency groups after controlling for all other determinants (*p* = 0.15). Children from low SES backgrounds reached their energy midpoint 12 min earlier than those from high SES families (*p* = 0.01), with a 9-min difference noted between middle and high SES groups (*p* = 0.02). Furthermore, children whose fathers were not working had a shorter fasting window (12 h) compared to those whose fathers worked regular hours (12 h 26 min) (*p* = 0.03). Additionally, children with fathers working regular hours exhibited a 12-min longer morning latency compared to children whose fathers worked shifts (*p* = 0.04). Age and sex did not show significant associations with any chrononutrition variables. Uni- and multilinear regression analyses supported the ANOVA and ANCOVA findings regarding the associations between continuous chronotype and age with chrononutrition variables (Fig. 1, Suppl. 1).

**Table 2:** Estimated marginal means (EMM), standard errors (SE) and *p* values for differences in chrononutrition variables across groups of potential determinants, 3-day mean values

Determinants	First eating occasion			Last eating occasion			Fasting window			Number of eating occasions			Morning latency			Evening latency			Eating midpoint			Energy midpoint		
	Model			Model			Model			Model			Model			Model			Model			Model		
	HH:MM	1	2	HH:MM	1	2	HH.MM	1	2		1	2	H.MM	1	2	H.MM	1	2	HH:MM	1	2	HH:MM	1	2
	EMM (SE)	*p* value		EMM (SE)	*p* value		EMM (SE)	*p* value		EMM (SE)	*p* value		EMM (SE)	*p* value		EMM (SE)	*p* value		EMM (SE)	*p* value		EMM (SE)	*p* value	
All	8:05 (04)			19:49 (04)			12.16 (05)			5.3 (0.07)			1.04 (04)			1.23 (04)			13:57 (03)			13:50 (04)		
Age		0.89	0.52		0.27	0.63		0.59	0.63		0.94	0.81		0.53	0.42		0.51	1.0		0.53	0.48		0.52	0.95
3–4 yrs	8:07 (04)			19:49 (04)			12.16 (05)			5.3 (0.07)			1.06 (04)			1.23 (04)			13:58 (03)			13:50 (04)		
5–6 yrs	8:05 (04)			19:48 (04)			12.18 (05)			5.3 (0.08)			1.03 (04)			1.23 (04)			13:56 (03)			13:50 (04)		
Sex		0.13	0.58		0.51	0.52		0.83	0.72		0.64	0.36		0.06	0.11		0.93	0.73		0.18	0.93		0.47	0.92
Boys	8:05 (04)			19:49 (04)			12.16 (05)			5.3 (0.08)			1.07 (04)			1.24 (04)			13:57 (03)			13:50 (04)		
Girls	8:07 (04)			19:47 (04)			12.17 (05)			5.3 (0.07)			1.02 (04)			1.23 (04)			13:57 (03)			13:50 (04)		
Chronotype tendency		< 0.001	< 0.001		< 0.001	< 0.001		0.66	0.98		0.02	0.15		< 0.001	0.002		< 0.001	< 0.001		< 0.001	< 0.001		< 0.001	< 0.001
Morning	7:35 (05)^a^			19:17 (06)^a^			12.17 (08)			5.4 (06)			1.19 (06)^a^			1.01 (06)^a^			13:26 (04)^a^			13:25 (06)^a^		
Intermediate	8:06 (03)^b^			19:50 (03)^b^			12.16 (04)			5.3 (04)			1.02 (04)^b^			1.22 (03)^b^			13:58 (02)^b^			13:54 (03)^b^		
Evening	8:36 (05)^c^			20:18 (06)^c^			12.17 (08)			5.1 (06)			0.52 (06)^b^			1.48 (05)^c^			14:27 (04)^c^			14:11 (06)^c^		
Mother work hours		0.07	0.16		0.23	0.25		0.01	0.10		0.82	0.85		0.19	0.40		0.50	0.60		0.94	0.91		0.67	0.61
Regular	8:02 (04)			19:52 (04)			12.10 (05)			5.3 (0.07)			1.01 (04)			1.23 (04)			13:57 (03)			13:51 (04)		
Shift work	8:10 (05)			19:47 (05)			12.21 (07)			5.3 (0.09)			1.08 (06)			1.26 (05)			13:58 (04)			13:52 (05)		
Do not work	8:06 (05)			19:46 (05)			12.19 (07)			5.2 (1.0)			1.05 (06)			1.21 (05)			13:56 (04)			13:47 (05)		
Father work hours		0.90	0.53		0.08	0.08		0.11	0.04		0.84	0.94		0.08	0.02		0.14	0.06		0.47	0.72		0.48	0.17
Regular	8:09 (03)			19:43 (03)			12.26 (04)^a^			5.3 (0.05)			1.13 (03)^a^			1.30 (03)			13:56 (02)			13:45 (03)		
Shift work	8:08 (05)			19:43 (05)			12.24 (07) ^ab^			5.3 (0.09)			1.01 (05)^b^			1.21 (05)			13:56 (04)			13:52 (05)		
Do not work	8:00 (07)			20:00 (08)			12.00 (10)^b^			5.3 (0.14)			0.59 (09)^ab^			1.19 (07)			14:00 (06)			13:53 (08)		
SES		0.72	0.81		0.04	0.54		0.05	0.34		0.36	0.56		0.23	0.62		0.47	0.49		0.38	0.95		< 0.001	0.004
Low	8:08 (04)			19:45 (05)			12.21 (06)			5.3 (0.05)			1.07 (05)			1.23 (05)			13:56 (04)			13:45 (05)^a^		
Middle	8:05 (04)			19:49 (04)			12.17 (06)			5.2 (0.05)			1.02 (05)			1.26 (04)			13:57 (03)			13:48 (04)^a^		
High	8:05 (04)			19:50 (04)			12.12 (06)			5.3 (0.05)			1.04 (05)			1.22 (04)			13:58 (03)			13:57 (04)^b^		

From the DAGIS study, 2015–2016

Model 1: One-way ANOVA was used to determine if there were significant differences among the groups. (n = 539–677), Model 2: ANCOVA was used to account for all independent determinants simultaneously as potential covariates in the analysis. (n = 503)

EMM: Represents the estimated marginal means adjusted for covariates. SE: standard error of the mean for the EMM. a,b,c: indicate statistically significant differences between groups. Groups with the same letter are not significantly different from each other.

Fasting window: time between last eating occasion one day and first eating occasion the following day. Morning/evening latency: time between waking up until first eating occasion/time between last eating occasion until falling asleep. Eating midpoint: time of day between first and last eating occasions. Energy midpoint: time of day when 50% of calories are consumed.

Chronotype tendency: natural preference for sleep and activity timing; Morning: earliest 10th chronotype percentile, Intermediate: 10–90th chronotype percentile, Evening tendency: latest 10th chronotype percentile.

SES: Socioeconomic Status; Low: comprehensive, vocational or high school education, Middle: bachelor’s degree or equivalent, High: master’s degree or licentiate/doctor.

#### Weekday and weekend results

Weekday results (Suppl. 2) were generally consistent with the 3-day mean findings, with some exceptions. An association was not identified between children with intermediate and evening chronotype tendencies regarding the timing of the energy midpoint, with a difference of seven minutes (*p* = 0.48). While the fathers’ work hours were not associated with the chrononutrition variables during weekdays, children of mothers with regular work hours had a 21-min shorter fasting window (*p* = 0.009) and their first EO earlier (7:41) (*p* = 0.006) compared to their peers whose mothers worked shifts (7:56).

On weekends no association was found between chronotype tendency and morning latency (*p* = 0.29) (Table 3); however, the difference between the sexes was significant, with boys displaying an 18-min longer morning latency compared to girls (*p* < 0.001). Regarding EOs, children with morning (*p* = 0.006) and intermediate (*P* = 0.02) chronotype tendencies had higher occurrences (5.4 and 5.1, respectively) compared to those with evening chronotype tendency (4.7). The associations between the fathers’ work hours and morning latency found in the 3-day mean results were also observed in the weekend results. In addition, children whose fathers were not working had significantly later timing for the last EO compared to children whose fathers worked regular hours (*p* = 0.02) or did shift work (*p* = 0.03). However, the mothers’ work hours showed no association with the chrononutrition variables during the weekend. SES showed only a borderline association with timing of the energy midpoint during the weekend (*p* = 0.05). Additionally, children aged 3–4 years had a shorter fasting window (12 h 12 min) compared to those aged 5–6 years (12 h 36 min) (*p* = 0.007), and boys had fewer EOs (5.0) compared to girls (5.2) (*p* = 0.03). In all other aspects, the findings for weekdays and weekends were largely consistent.

**Table 3:** Estimated marginal means (EMM), standard errors (SE) and *p* values for differences in chrononutrition variables across groups of potential determinants during the weekend from the DAGIS study, 2015–2016

Determinants	First eating occasion			Last eating occasion			Fasting window			Number of eating occasions			Morning latency			Evening latency			Eating midpoint			Energy midpoint		
	Model			Model			Model			Model			Model			Model				Model			Model	
	HH:MM	1	2	HH:MM	1	2	HH.MM	1	2		1	2	H.MM	1	2	H.MM	1	2	HH:MM	1	2	HH:MM	1	2
	EMM (SE)	*p* value		EMM (SE)	*p* value		EMM (SE)	*p* value		EMM (SE)	*p* value		EMM (SE)	*p* value		EMM (SE)	*p* value		EMM (SE)	*p* value		EMM (SE)	*p* value	
All	8:38 (05)			20:05 (07)			12.24 (12)			5.1 (1.2)			1.27 (06)			1.22 (10)			14:16 (06)			14:13 (06)		
Age		0.43	0.42		0.52	0.64		0.18	0.007		0.43	0.53		0.78	0.59		0.23	0.81		0.22	0.26		0.15	0.39
3–4 yrs	8:37 (06)			20:06 (08)			12.12 (13) ^a^			5.1 (1.3)			1.26 (07)			1.23 (11)			14:14 (06)			14:11 (06)		
5–6 yrs	8:40 (06)			20:04 (08)			12.36 (13) ^b^			5.0 (1.3)			1.29 (07)			1.21 (11)			14:19 (06)			14:15 (06)		
Sex		0.65	0.42		0.52	0.51		0.57	0.52		0.07	0.03		< 0.001	< 0.001		0.78	0.66		0.25	0.72		0.98	0.83
Boys	8:40 (06)			20:07 (08)			12.27 (13)			5.0 (1.3) ^a^			1.36 (07) ^a^			1.24 (11)			14:17 (06)			14:13 (06)		
Girls	8:37 (06)			20:03 (08)			12.21 (13)			5.2 (1.3) ^b^			1.18 (07) ^b^			1.21 (11)			14:16 (06)			14:12 (06)		
Chronotype tendency		< 0.001	< 0.001		< 0.001	< 0.001		0.72	0.54		0.01	0.006		0.55	0.29		< 0.001	< 0.001		< 0.001	< 0.001		< 0.001	< 0.001
Morning	7:49 (08)^a^			19:30 (11)^a^			12.12 (17)			5.4 (1.8)^a^			1.30 (10)			0.42 (16)^a^			13:33 (08)^a^			13:31 (09)^a^		
Intermediate	8:34 (05)^b^			20:05 (07)^b^			12.28 (10)			5.1 (1.1)^a^			1.20 (06)			1.16 (09)^b^			14:18 (05)^b^			14:13 (05)^b^		
Evening	9:32 (08)^c^			20:39 (11)^c^			12.32 (18)			4.7 (1.8)^b^			1.32 (10)			2.09 (15)^c^			14:58 (08)^c^			14:54 (09)^c^		
Mother’s work hours		0.66	0.59		0.37	0.37		0.58	0.77		0.52	0.95		0.83	0.77		0.39	0.52		0.57	0.17		0.24	0.05
Regular	8:41 (05)			20:11 (08)			12.26 (12)			5.0 (1.2)			1.30 (07)			1.20 (10)			14:22 (06)			14:20 (06)		
Shift work	8:35 (07)			20:03 (10)			12.28 (15)			5.1 (1.6)			1.24 (09)			1.15 (14)			14:16 (07)			14:15 (08)		
Do not work	8:49 (07)			20:00 (10)			12.17 (16)			5.1 (1.6)			1.27 (09)			1.32 (14)			14:11 (07)			14:03 (08)		
Father’s work hours		0.82	0.67		0.20	.03		0.21	0.05		0.70	0.73		0.04	0.03		0.36	0.09		0.50	0.32		0.42	0.39
Regular	8:42 (04)			19:50 (05)^a^			12.41 (08)			5.0 (.9)			1.41 (05)			1.41 (08)			14:09 (04)			14:06 (04)		
Shift work	8:41 (05)			19:49 (08)^a^			12.52 (13)			5.0 (1.6)			1.25 (09)			1.35 (11)			14:16 (06)			14:11 (07)		
Do not work	8:32 (11)			20:36 (17)^b^			11.39 (28)			5.2 (2.5)			1.15 (14)			0.50 (24)			14:24 (13)			14:20 (12)		
SES		0.31	0.69		0.91	0.96		0.56	0.99		0.45	0.78		0.10	0.71		0.59	0.70		0.48	0.55		0.002	0.05
Low	8:41 (07)			20:06 (09)			12.24 (14)			5.0 (1.5)			1.31 (08)			1.23 (13)			14:19 (07)			14:06 (07)		
Middle	8:38 (06)			20:05 (08)			12.23 (14)			5.1 (1.3)			1.25 (07)			1.26 (12)			14:13 (06)			14:11 (06)		
High	8:36 (06)			20:04 (09)			12.24 (14)			5.1 (1.4)			1.25 (08)			1.18 (12)			14:16 (06)			14:21 (07)		

Model 1: One-way ANOVA was used to determine if there were significant differences among the groups. (n = 312–675), Model 2: ANCOVA was used to account for all independent determinants simultaneously as potential covariates in the analysis. (n = 231–501)

EMM: Represents the estimated marginal means adjusted for covariates. SE: standard error of the mean for the EMM. a,b,c: indicate statistically significant differences between groups. Groups with the same letter are not significantly different from each other.

Fasting window: time between last eating occasion one day and first eating occasion the following day. Morning/evening latency: time between waking up until first eating occasion/time between last eating occasion until falling asleep. Eating midpoint: time of day between first and last eating occasions. Energy midpoint: time of day when 50% of calories are consumed. Chronotype tendency: natural preference for sleep and activity timing; Morning: earliest 10th chronotype percentile, Intermediate: 10–90th chronotype percentile, Evening tendency: latest 10th chronotype percentile.

SES: Socioeconomic Status; Low: comprehensive, vocational or high school education, Middle: bachelor’s degree or equivalent, High: master’s degree or licentiate/doctor.

## Discussion

The present study explored the associations between chronotype tendency, SES, parents’ work hours, age, and sex as potential determinants of various chrononutrition factors in Finnish preschool children aged 3–6 years. Among the examined determinants, chronotype tendency was found to have the most substantial associations with key outcomes including the timing of first and last EOs, the duration of the fasting window, morning and evening latency, the number of EOs, and the eating and energy midpoints. In comparison, SES, parents’ work hours, age, and sex showed both weaker and fewer associations with the same outcomes. Weekday results largely aligned with the 3-day mean findings, with a few notable differences. No association was observed between children with intermediate and evening chronotype tendencies in terms of the energy midpoint timing during weekdays. Additionally, maternal work hours were associated with chrononutrition variables only during weekdays, while fathers’ work hours were associated only during weekend days. On weekends, differences in the number of EOs and duration of morning latency were significant between boys and girls.

### Chronotype

Chronotype tendency showed an association with several chrononutrition variables. Individuals with an earlier chronotype had earlier timings for their first and last EOs, as well as earlier eating and energy midpoints. This finding aligns with previous results from a cross-sectional study conducted on school-aged children in China [23]. Contrary to an earlier study conducted on 496 children aged 7–11 years in Hong Kong, which indicated that children with evening chronotype tendency are more likely to skip breakfast, leading to longer morning latency [24], our study found that during weekdays, children with morning chronotype tendencies exhibited the longest morning latency while children with evening chronotype tendencies had the shortest morning latency. One explanation could be that the children in our study were younger than in the previous research and therefore had less autonomy regarding timing. No association was found between chronotype tendency and the duration of morning latency on weekend days in the current study. This suggests that the shorter morning latency on weekdays observed among children with later chronotype tendencies may be attributed to a structured preschool schedule, which leaves them with less time between waking up and having their first EO compared to children with earlier chronotype tendencies. In accordance with results in the previous studies conducted in Asian children aged 7–12 years [23, 24], children with morning chronotype tendencies in our study had the earliest first EO, while children with evening chronotype tendencies had the latest first EO. In agreement with the earlier findings in older children [23, 24], children with evening chronotype tendencies in this study had their last EO an hour later in the day than children with morning chronotype tendencies. Although later eating times are generally assumed to shorten the fasting window [45], this was not evident in our sample, as no significant difference in the duration of the fasting window was observed between the groups on neither weekdays nor weekends. This discrepancy may be explained by the fact that children with an evening chronotype tendency experienced longer evening latency compared to their morning counterparts, averaging a difference of 31 min on weekdays and 1 h and 27 min on weekends.

Children identified as having a morning chronotype tendency tend to consume a greater proportion of their daily energy intake earlier in the day [30]. This aligns with our results, which showed that children with morning chronotype tendencies achieved both their energy and eating midpoints earlier than their evening counterparts. The differences in eating timing between chronotypes are often more pronounced on weekends, when individuals are not bound by structured schedules, such as preschool commitments, allowing families to follow their natural preferences [21]. A previous review concluded that adults with a morning chronotype typically exhibit a more consistent eating schedule, while evening chronotypes tend to shift their eating habits later on weekends compared to weekdays [2]. Similarly, the current study observed a notable shift in eating behaviour on weekends, with pronounced differences between chronotype tendencies regarding first and last EOs, energy midpoint, and evening latency. Additionally, it was found that children with evening chronotype tendencies had fewer EOs during the weekend compared to children with intermediate or morning chronotype tendencies, although this pattern did not hold during the week. This phenomenon may be attributed to children with evening chronotype tendencies waking up later on weekends [2], which may lead to later energy intake. Consequently, these individuals may not feel hungry in the morning, making them more likely to skip breakfast and lunch, thereby resulting in fewer EOs overall [8, 46]. Nonetheless, it is important to consider the role of parents, as young children may lack the autonomy to decide whether to skip EOs.

### SES and parents’ work hours

Children from high SES backgrounds exhibited later energy midpoints on both weekdays and weekends, while other chrononutrition variables showed no notable differences between the SES groups. Higher SES families may have more structured daily schedules with later dinner times, aligning with parental work hours and children’s extracurricular activities. An Australian online study on children aged 6 months to 6 years, found that eating dinner later was not associated with high SES, but the parents rated the importance of family meals higher and also had a higher frequency of family meals [47]. Hence, the family mealtime environments and behaviours may impact the children’s energy intake at mealtimes. Children from lower SES backgrounds tend to rely more on meals provided at preschool, with ECEC meals appearing to help balance differences in dietary quality between children of different socioeconomic groups (DAGIS, unpublished result). Most research concerning children has primarily concentrated on SES’s association with nutritional intake [1, 38, 48], rather than on the timing of food consumption. Nonetheless, children with lower SES have been linked to higher likelihood of skipping meals, particularly breakfast, when compared to their higher SES counterparts [49, 50]. This was not observed in the current study. A possible explanation would be that, in addition to lunch and snacks, breakfast is provided free of charge at ECEC centers in Finland.

Parents’ work hours were modestly associated with the chrononutrition variables in this study. Mothers’ work hours were more influential during the week, while fathers’ hours had a stronger association with the chrononutrition patterns on the weekend. Several studies have shown that fathers spend more time with their children during weekends compared to weekdays [51, 52], and therefore, might have a bigger impact on the timing of food intake on free days. A population-based cohort in Taiwan found that preschool-aged children whose parents worked nonstandard hours were less likely to eat breakfast regularly [22], which is in accordance with our findings, where children with mothers working shifts were associated with later first EOs and a longer fasting window on weekdays compared to children with mothers working regular hours. Parents’ shift work tends to disrupt schedules more during weekdays because of the set routines e.g., preschool, while families have more flexibility during the weekend [53]. During the weekend days, children with nonworking fathers had later last EOs compared to those with working fathers, possibly due to more flexible routines.

### Age and sex

Results from the present study indicated that age and sex were only associated with differences in the chrononutrition variables observed on weekend days. Children in the younger age group had a shorter fasting window, on average 12h12min compared to children in the older age group whose fasting window on average lasted 12h36min. An explanation for the longer fasting window duration in the older children is a potentially longer sleep duration on weekend nights compared to the younger age group children. By age 5, 94% of children no longer take naps [54]; thus, more participants in the younger age group may still be napping daily, leading to fewer hours of night time sleep and affecting the duration of the nightly fasting window [55].

This study found no differences between the sexes during weekdays, but on weekend days, boys had a longer morning latency and fewer EOs compared to girls. No previous research on preschool-aged children comparing the sexes regarding chrononutrition factors was found. However, a Polish study on preschool-aged children found that parents were more concerned with managing their daughters’ overall eating habits than their sons’ [56], perhaps explaining their shorter morning latency in the current study. An observational study in the U.S conducted on kindergarteners showed that girls tend to seek parental approval more often and are praised more frequently than boys by their mothers when eating [57]. Boys might prefer to engage in other activities after waking up, such as playing or using electronic devices, which might delay their first EO and lead to fewer EOs overall. More research is needed to draw conclusions about differences between the sexes regarding morning latency and the frequency of EOs, as skipping breakfast and a lower number of EOs are associated with overweight [12, 58], and studies show that overweight is more prevalent in boys compared to girls [59, 60].

### Strengths and limitations

It is important to recognize certain limitations inherent in this study. First, the families participating in this study had higher levels of education compared to national averages [61], potentially impacting the generalizability to our findings. All participants attended preschool, so the results might differ for children cared for at home. However, since the majority (78.6%) of Finnish children aged 3–5 attend preschool [62], the generalizability of the findings remains largely intact. Second, the cut-off scores for chronotype tendencies in this study are specific to our sample, as participants were grouped based on the lowest and highest 10th percentiles, a method previously used in the DAGIS study [63]. While this approach assumes a normal distribution of chronotypes similar to the broader population, the cut-off scores might not align with those of the entire population of children the same age. Furthermore, with conservative prevalence estimates set at 10%, there is a possibility that some children with morning or evening chronotype tendencies were categorized into the intermediate chronotype group. However, through this approach, we most likely captured true positives in the morning and evening chronotype tendency groups, and in addition, the analyses were also conducted with chronotype as a continuous variable. While weekend night sleep data were utilized to determine chronotype, we cannot guarantee that children were not awakened on weekend mornings for example due to scheduled activities such as hobbies. Additionally, we did not collect data on parental chronotypes, which may influence the children’s schedules for timing of sleep and food intake. Third, the study’s cross-sectional nature means that causality cannot be concluded from the findings, as it only captures data at a single point in time.

To our knowledge no previous research has examined the determinants of chrononutrition factors in preschool aged children. The strengths of the current study include its relatively large sample situated in both rural and urban areas of Finland. Participants’ parents and preschool personnel recorded food and beverage intake in real time, while actigraphy was used to measure sleep, reducing recall bias. Food records were collected on the same dates as three of the 7 days of actigraphy data, encompassing both weekdays and weekends. This simultaneous data collection provided a more accurate representation of chrononutrition variables. Including both weekdays and weekends allowed for a comprehensive view of how the determinants of chrononutrition patterns varied between weekdays and weekends.

## Conclusion

Our findings reveal that chronotype tendency played a significant role in shaping the chrononutrition patterns of Finnish preschool-aged children. In contrast, SES, parents’ work hours, age, and sex were associated to a lesser extent with the timing of food intake. This study addresses an important gap in our understanding of the potential determinants of chrononutrition patterns during early childhood, offering new insights into the factors related to children’s timing of food intake. The information yielded from this research can be used when developing effective interventions and public health strategies aimed at preventing obesity, enhancing metabolic health, and promoting overall healthy eating habits. Further research, particularly longitudinal studies, is essential to deepen our understanding of the factors associated with the timing and regularity of food intake, and how it relates to the nutritional intake of preschool-aged children.

## Supplementary Information

Below is the link to the electronic supplementary material.

Supplementary file1 (PDF 262 KB)

## Data Availability

The datasets produced and/or analysed in this study are not publicly accessible due to ethical and privacy constraints. However, they can be obtained from the principal investigator of the DAGIS Survey (ER).
