# Supplementary material for: Associations of chronotype and socio-demographic factors with timing of eating in finnish preschool-aged children
Source: Eur J Nutr. 2025 Sep 18;64(6):279. doi: 10.1007/s00394-025-03800-z (PMC12446394; doi:10.1007/s00394-025-03800-z)
Supplement: Supplementary file 1 — Supplementary file1 (PDF 262 KB) [file 394_2025_3800_MOESM1_ESM.pdf]

Associations of Chronotype and Socio-Demographic Factors with Timing of Eating in Finnish Preschool-Aged Children
European Journal of Nutrition

Ilse Tillman[1,2], Mirkka Maukonen[2,3], Anna M Abdollahi[1], Henna Vepsäläinen[1], Carola Ray[1,2], Jenna Rahkola[2], Eva Roos[2,4,5], Maijaliisa Erkkola[1], Reetta Lehto[1,2]

1 Department of Food and Nutrition, University of Helsinki, Finland.
2 Folkhälsan Research Center, Helsinki, Finland.
3 Finnish Institute for Health and Welfare, Finland.
4 Department of food studies, Nutrition and Dietetics, Uppsala University, Uppsala, Sweden.
5 Department of Public Health, University of Helsinki, Finland.

Corresponding author
Ilse Tillman
ilse.tillman@helsinki.fi

Suppl. 1 Linear Regression for 3-Day Mean Values. From the DAGIS study 2015-2016.

	Age						Chronotype					
	Univariate Model			Multivariate Model			Univariate Model			Multivariate Model		
**Dependent Variables**	**ß (SE)**	**(95%) CI**	**p-value**	**ß (SE)**	**(95%) CI**	**p-value**	**ß (SE)**	**(95%) CI**	**p-value**	**ß (SE)**	**(95%) CI**	**p-value**
**First Eating Occasion**	.01 (.03)	-.04, .06	.59	-.01 (.02)	-.05, .04	.80	.37 (.03)	.31, .43	<.001	.39 (.04)	.32, .46	<.001
**Last Eating Occasion**	.05 (.03)	-.00, .10	.06	-.00 (.02)	-.05, .04	.90	.48 (.03)	.41, .54	<.001	.48 (.04)	.41, .55	<.001
**Fasting Window**	-.02 (.03)	-.08, .05	.56	.02 (.03)	-.05, .09	.62	-.09 (.04)	-.17 -.00	.05	-.06 (.05)	-.17, .04	.24
**Number of Eating Occasions**	-.02 (.03)	-.08, .03	.37	-.02 (.03)	-.08, .03	.41	-.09 (.04)	-.16, -.02	.01	-.07 (.04)	-.15, .02	.14
**Morning Latency**	-.01 (.03)	-.07, .04	.71	-.01 (.03)	-.06, .05	.74	-.23 (.04)	-.30, -.16	<.001	-.23 (.04)	-.31, -.14	<.001
**Evening Latency**	-.01 (.03)	-.06, .04	.57	-.02 (.02)	-.06, .03	.54	.28 (.03)	.22, .35	<.001	.29 (.04)	.21, .36	<.001
**Eating Midpoint**	.03 (.02)	-.01, .07	.13	-.00 (.02)	-.04, .03	.80	.42 (.02)	.38, .47	<.001	.44 (.03)	.38, .49	<.001
**Energy Midpoint**	.05 (.03)	-.00, .10	.06	.02 (.03)	-.03, .07	.47	.33 (.03)	.27, .40	<.001	.32 (.04)	.25, .40	<.001

Fasting window: Time between last eating occasion one day and first eating occasion the following day. **Morning/evening latency:** Time between waking up until first eating occasion/time between last eating occasion until falling asleep. **Eating midpoint:** Time of day between first and last eating occasions. **Energy midpoint:** Time of day when 50% of calories are consumed.
Chronotype: As continuous variable. Natural preference for sleep and activity timing.

Suppl. 2 Estimated Marginal Means (EMM), Standard Errors (SE) and p-values for Chrononutrition Variables Across the Determinants During Weekdays. From the DAGIS Study, 2015-2016

Determinants	First Eating Occasion			Last Eating Occasion			Fasting Window			Number of Eating Occasions		
		Model			Model			Model			Model	
	HH:MM	1	2	HH:MM	1	2	HH.MM	1	2		1	2
	EMM (SE)	p-value		EMM (SE)	p-value		EMM (SE)	p-value		EMM (SE)	p-value	
**All**	7:49 (04)			19:44 (04)			12.04 (05)			5.3 (.8)		
**Age**		.42	.13		.52	.58		.20	.37		.25	.19
3-4 yrs.	7:51 (04)			19:45 (04)			12.06 (06)			5.3 (.8)		
5-6 yrs.	7:46 (04)			19:43 (04)			12.02 (06)			5.4 (.8)		
**Sex**		.0.9	.23		.97	.61		.13	.17		.90	.95
Boys	7:47 (04)			19:45 (04)			12.01 (06)			5.3 (.8)		
Girls	7:51 (04)			19:43 (04)			12.08 (06)			5.3 (.8)		
**Chronotype**		<.001	<.001		<.001	<.001		.23	.42		.12	.61
Morning	7:28 (06) [a]			19:14 (06) [a]			12.11 (09)			5.4 (1.2)		
Intermediate	7:52 (03) [b]			19:47 (03) [b]			12.05 (05)			5.4 (.7)		
Evening	8:07 (06) [c]			20:11 (06) [c]			11.57 (08)			5.3 (1.2)		
**Mother's Work Hours**		.001	.005		.31	.45		.004	.009		.39	.71
Regular	7:41 (04) [a]			19:47 (04)			11.54 (05) [a]			5.4 (.8)		
Shift Work	7:56 (05) [b]			19:40 (05)			12.15 (07) [b]			5.3 (1.1)		
Do Not Work	7:49 (06) [a,b]			19:45 (06)			12.04 (08) [a,b]			5.3 (1.1)		
**Father's Work Hours**		.94	.68		.28	.29		.33	.22		.94	.99
Regular	7:52 (03)			19:39 (03)			12.12 (04)			5.3 (.6)		
Shift Work	7:51 (05)			19:41 (05)			12.09 (07)			5.3 (1.0)		
Do Not Work	7:44 (08)			19:52 (08)			11.52 (11)			5.3 (1.6)		
**SES**		.91	.95		.26	.10		.05	.19		.31	.45
Low	7:50 (05)			19:38 (05)			12.12 (07)			5.3 (1.0)		
Middle	7:49 (04)			19:48 (04)			12.01 (06)			5.3 (.9)		
High	7:48 (05)			19:46 (05)			12.01 (06)			5.4 (.9)		

Determinants	Morning Latency			Evening Latency			Eating Midpoint			Energy Midpoint		
		Model			Model			Model			Model	
	H.MM	1	2	H.MM	1	2	HH:MM	1	2	HH:MM	1	2
	EMM (SE)	p-value		EMM (SE)	p-value		EMM (SE)	p-value		EMM (SE)	p-value	
**All**	0.52 (04)			1.24 (04)			13:46 (03)			13:41 (04)		
**Age**		.22	.08		.31	.68		.92	.16		.96	.70
3-4 yrs.	0.55 (05)			1.25 (04)			13:48 (03)			13:42 (05)		
5-6 yrs.	0.48 (05)			1.23 (05)			13:45 (03)			13:40 (05)		
**Sex**		.90	.70		.88	.96		.28	.64		.44	.80
Boys	0.51 (05)			1.24 (05)			13:46 (03)			13:40 (05)		
Girls	0.52 (05)			1.24 (04)			13:47 (03)			13:41 (05)		
**Chronotype**		<.001	<.001		<.001	<.001		<.001	<.001		<.001	<.001
Morning	1.13 (07) [a]			1.08 (06) [a]			13:21 (05) [a]			13:23 (07) [a]		
Intermediate	0.52 (04) [b]			1.25 (04) [b,c]			13:49 (03) [b]			13:46 (04) [b,c]		
Evening	0.30 (07) [c]			1.39 (07) [c]			14:09 (04)[c]			13:53 (07) [c]		
**Mother's Work Hours**		.06	.06		.15	.14		.53	.34		.99	.70
Regular	0.45 (04)			1.25 (04)			13:44 (03)			13:39 (04)		
Shift Work	0.58 (06)			1.30 (06)			13:48 (04)			13:43 (06)		
Do Not Work	0.51 (06)			1.17 (06)			13:47 (04)			13:41 (06)		
**Father's Work Hours**		.33	.07		.59	.40		.69	.89		.65	.34
Regular	0.58 (03)			1.27 (03)			13:45 (02)			13:36 (03)		
Shift Work	0.46 (05)			1.19 (06)			13:46 (04)			13:44 (06)		
Do Not Work	0.50 (09)			1.26 (09)			13:48 (06)			13:42 (09)		
**SES**		.44	.95		.47	.39		.26	.41		.02	.02
Low	0.52 (06)			1.27 (05)			13:44 (04)			13:36 (05) [a]		
Middle	0.51 (05)			1.25 (05)			13:48 (03)			13:38 (05) [a]		
High	0.51 (05)			1.20 (05)			13:47 (03)			13:48 (05) [b]		

Model 1: One-way ANOVA was used to determine if there were significant differences among the groups. (n =661-677), **Model 2**: ANCOVA was used to account for all independent determinants simultaneously as potential covariates in the analysis. (n = 491-503)

EMM: Represents the Estimated Marginal Means adjusted for covariates. **SE**: Standard Error of the Mean for the EMM. **a,b,c**: indicate statistically significant differences between groups. Groups with the same letter are not significantly different from each other.

Fasting window: Time between last eating occasion one day and first eating occasion the following day. **Morning/evening latency:** Time between waking up until first eating occasion/time between last eating occasion until falling asleep. **Eating midpoint:** Time of day between first and last eating occasions. **Energy midpoint:** Time of day when 50% of calories are consumed.

Chronotype tendency: Natural preference for sleep and activity timing; Morning: earliest 10th chronotype percentile, Intermediate: 10–90th chronotype percentile, Evening tendency: latest 10th chronotype percentile.

SES: Socioeconomic Status; Low: comprehensive, vocational or high school education, Middle: bachelor's degree or equivalent, High: master's degree or licentiate/doctor.
